# Practical method to determine the effective zero-point of indentation depth for continuous stiffness measurement nanoindentation test with Berkovich tip

**DOI:** 10.1038/s41598-022-10490-8

**Published:** 2022-04-16

**Authors:** Diancheng Geng, Hao Yu, Yasuki Okuno, Sosuke Kondo, Ryuta Kasada

**Affiliations:** 1 Institute for Materials Research, Tohoku University, Sendai, Miyagi 980-8577 Japan; 2 Department of Quantum Science and Energy Engineering, Graduate School of Engineering, Tohoku University, Sendai, Miyagi 980-8579 Japan

**Keywords:** Structural materials, Techniques and instrumentation

## Abstract

The zero-point of indentation depth in nanoindentation or depth-sensing instrumented indentation tests should be precisely set to evaluate the indentation hardness and indentation elastic modulus of materials to be tested, especially at shallow depths. A critical contact stiffness value has been widely used to determine the zero-point in nanoindentation tests with a Berkovich tip using the continuous stiffness measurement (CSM) method. However, this criterion occasionally gives an inadequate zero-point owing to the surface roughness of materials, the vibration of the testing system, and the flaws of the CSM method at shallow depth. This study proposes a practical method to determine the effective zero-point of indentation depth, which was obtained linearly at the zero-point of contact stiffness and extrapolated from the depth-dependent contact stiffness values, except for those at initially unstable contact depths. The proposed method enables nanoindentation tests to obtain a constant indentation elastic modulus and low deviation of nanoindentation hardness of homogenously fused silica and metallic materials, which provides an efficient way to obtain more accurate test data.

## Introduction

Nanoindentation or depth-sensing instrumented indentation tests dominate in measuring the mechanical properties of size-limited materials, such as thin foil, coating, and depth-dependent materials, for many technological and biomedical applications^1–5^. The continuous stiffness measurement (CSM) method, which can continuously obtain the depth-dependent contact stiffness through one indent, can evaluate the indentation elastic modulus and hardness as a function of indentation depth^6–10^. The determination of the zero-point of indentation depth as the first contact of the tip onto the sample surface has received attention for a long time because the zero-point has always been one of the sources of potential errors^9,11,12^. Although the zero-point should correspond to the first contact between the sample surface and the tip, it can be affected by the conditions of the sample and tip simultaneously.

A typical zero-point determination by the CSM method using a critical value of contact stiffness can cause a significant error in the results. Additionally, the automatic determination of zero-point by the critical value of contact stiffness can differ significantly from the real value owing to environmental factors, such as the vibration and surface conditions of the materials to be tested. A theoretical approach based on the Hertzian contact was used for spherical indenter with the CSM method to find the effective zero-point^13^, de-emphasising artefacts owing to unfavourable surface conditions. However, the Hertzian contact condition is not useful for indentation by a sharp Berkovich tip, which has been commonly used to obtain indentation elastic modulus and hardness because it ideally undergoes plastic deformation while practically achieving elastic contact only for the rounded tip with a limited elastic deformed zone in the indented material. Therefore, developing an effective zero-point determination method for the nanoindentation test using the CSM method with a Berkovich tip is necessary. This study introduces a practical determination method for the effective zero-point of contact by linearly extrapolating the values of contact stiffness obtained by the CSM method with a Berkovich indenter. Consequently, the stable indentation elastic modulus and indentation hardness of fused silica and metallic materials such as Cu, Fe, and W were obtained through zero-point adjustment. The errors in the nanoindentation results due to the zero-point shift were examined to elucidate their impact on the indentation elastic modulus and hardness as well as their depth dependences at shallow depths.

## Theory

In the nanoindentation tests^14^, the reduced elastic modulus ${E}^{*}$ can be obtained as

1 $${E}^{*}=\frac{1}{2\beta }S\frac{\sqrt{\pi }}{\sqrt{A}}$$

where $A$ represents the projected area of contact, and $\beta$ denotes a constant that depends on the geometry of the indenter ($\beta =1.034$ for a Berkovich indenter^15^). Generally, the contact stiffness $S$ is extracted from the elastic unloading, which is the initial part of the unloading curve, while it can be alternatively measured during the loading portion of an indentation test using the CSM technique^16^. The reduced elastic modulus ${E}^{*}$ can be expressed as follows:

2 $$\frac{1}{{E}^{*}}=\frac{(1-{\nu }_{s}^{2})}{{E}_{s}}+\frac{(1-{\nu }_{i}^{2})}{{E}_{i}}$$

where $E$ and $v$ represent the elastic modulus and Poisson's ratio corresponding to diamond indenter *i* and sample $s$, respectively. As a geometrically similar indenter, the ideal projected contact area $A({h}_{c})$ of the Berkovich tip can be expressed as follows:

3 $$A({h}_{c})=24.50\times {{h}_{c}}^{2}$$

where ${h}_{c}$ denotes the contact depth. Combining Eqs. (1) and (3), we obtain the following:

4 $$S=2\beta {E}^{*}\sqrt{\frac{24.5}{\pi }}\times {h}_{c}$$

In dimensional analysis, the contact depth can be obtained as^17^

5 $${h}_{c}=h{\prod }_{\beta }\left(\frac{Y}{E},v,n,\theta \right)$$

where ${\prod }_{\beta }={h}_{c}/h$. Furthermore, $Y$, $E$, $v$, $n$, and $\theta$ are the yield stress, elastic modulus, Poisson’s ratio, strain-hardening exponent, and indenter geometry, respectively. The linear dependence between ${h}_{c}$ and $h$ was verified by finite element calculations for a wide range of values of $Y$, $E$, and $n$ ^18^. The linear relationship between ${h}_{c}$ and $h$ was also verified by Li et al^16^, Zhu et al.^19^ and Gao et al^20,21^.

Therefore, Eq. 4 can be written as

6 $$S=2\beta {E}^{*}\sqrt{\frac{24.5}{\pi }}{\prod }_{\beta }\left(\frac{Y}{E},v,n,\theta \right)\times h$$

Considering the zero-point shift in practice with Eqs. (1), (3), and ${H}_{IT}=P/A$, where $P$ represents the load applied to the tip, the errors in indentation elastic modulus and hardness can be derived as

7 $$ \frac{{\Delta E_{IT} }}{{E_{IT} }} = \frac{{E_{IT}^{^{\prime}} - E_{IT} }}{{E_{IT} }} = \frac{{\sqrt A - \sqrt {A^{{\prime }} } }}{{\sqrt {A^{\prime}} - \frac{{\left( {1 - \nu_{i}^{2} } \right)}}{{E_{i} }} \times \frac{\sqrt \pi \times S}{{2\beta }}}} = \frac{{\sqrt A - \sqrt {A^{{\prime }} } }}{{\sqrt {A^{\prime}} - 0.00074 \times S}}\;{\text{and}} $$

8 $$  \frac{{\Delta H_{{IT}} }}{{H_{{IT}} }} = \frac{{H_{{IT}}^{{\prime }}  - H_{{IT}} }}{{H_{{IT}} }} = \frac{{A - A^{{\prime }} }}{{A^{{\prime }} }} = \frac{{h_{c} ^{2}  - h_{c}^{{'2}} }}{{h_{c}^{{{\prime }2}} }} =  - \frac{{\frac{{\Delta h_{c} }}{{h_{c} }}\left( {\frac{{\Delta h_{c} }}{{h_{c} }} + 2} \right)}}{{\left( {\frac{{\Delta h_{c} }}{{h_{c} }} + 1} \right)^{2} }},~\;{\text{where}}\;\;\Delta h_{c}  = h_{c}^{{\prime }}  - h_{c}  $$

Symbols with prime denote the parameters after zero-point shift.

**Figure 1:**
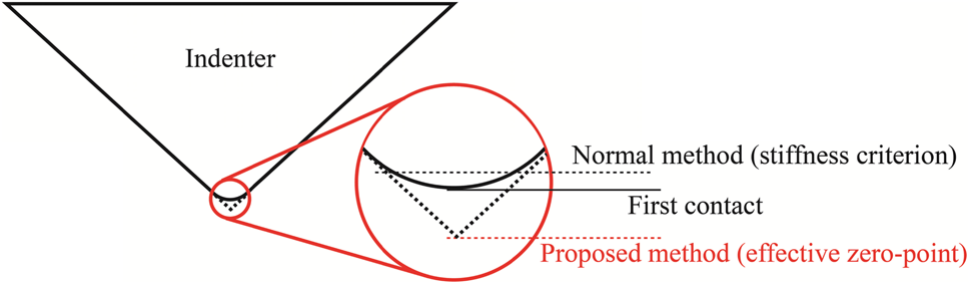
Difference in zero-point determination between the normal (stiffness criterion) and proposed methods.

The contact stiffness *S* of the Berkovich indenter on homogenous materials is expected to be a linear function against the indentation depth ℎ, implying that this linear function can be a criterion to find the effective zero-point with the modified Berkovich indenter. However, it should be noted that the initial contact of the practical Berkovich tip is elastic owing to the tip rounding. The surface roughness leads to the zero-point shift, and the tip rounding enlarges the difference between the first contact and the effective zero-point. In addition to the problems mentioned above, losing contact tapping in the CSM method^22^ would inevitably fail the regular zero-point determination. Therefore, the normal zero-point determination method and even the present linear relationship between *S* and *h* (Eq. 6) cannot be applied at the beginning of the increasing contact stiffness. The present study performed a linear fitting to the contact stiffness values from the stable elastic–plastic contact point. The different zero-point definitions of these methods are schematically shown in Fig. 1. The normal method generally estimates the zero-point to be deeper than the actual first contact, which results in a larger contact area than the actual one. In contrast, the present method estimates the zero-point to be shallower than the actual first contact, but the zero-point corresponds to the ideal sharp tip.

Indentation size effect (ISE), which is a decrease in hardness with the increase in indentation depth, is often observed for various materials in nanoindentation hardness. The Nix–Gao model^23^, based on the concept of geometrically necessary dislocations formed around the indentation imprint, has been widely accepted and explains the ISE as

9 $$H={H}_{0}\sqrt{1+\frac{{h}^{*}}{h}}$$

where ${H}_{0}$ and ${h}^{*}$ represent the bulk-equivalent hardness corresponding to the infinite depth and the length that characterizes the depth dependence of the hardness, respectively. While recent literature recommends that the CSM method should not be used for the study of ISE because of the uncertainty of hardness data at shallow depth^22^, exploring the effect of zero-point adjustment on the ISE is considered to be valuable for understanding the ISE.

## Results

### Zero-point adjustment on standard fused silica

**Figure 2:**
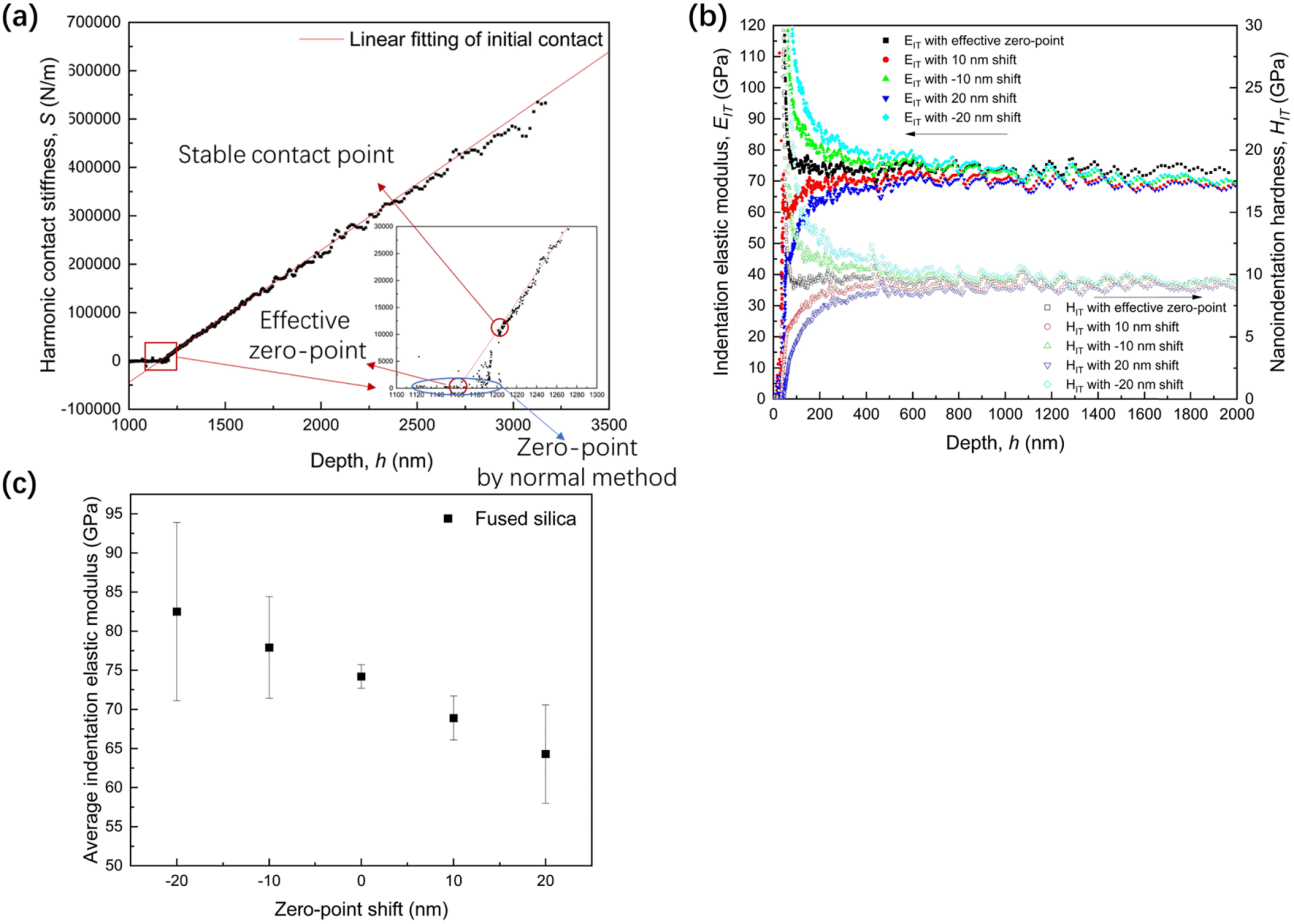
Zero-point adjustment and results on fused silica, and the impact of the zero-point shift on indentation elastic modulus. (**a**) Linear fitting and extrapolation on nanoindentation data to determine the effective zero-point. Red circle and blue circle show the possible zero-point by proposed method and stiffness criterion, respectively. (**b**) Indentation elastic modulus results after zero-point adjustment. (**c**) Indentation elastic modulus and nanoindentation hardness after zero-point shift. (**d**) Average and standard deviation of indentation elastic modulus after zero-point shift.

Prior to the main measurements, the tip area function must be obtained with a correct zero-point from the results of standard materials, such as fused silica. Figure 2a shows a typical example of the contact stiffness variation in fused silica against the raw data of indentation depth. As shown in Fig. 2a, the initial zero-point determined by the stiffness criterion is easily affected by the environmental vibration. Ignoring this extreme error, the zero-point is re-determined by the normal method using a stiffness criterion, which is 200 N/m in this case. However, it is difficult to obtain only one zero-point using the normal method owing to the unstable contact of the rounded tip with the material surface having roughness. The possible range of zero-points detected by the stiffness criterion could exceed 200 nm as shown in the blue circle in Fig. 2a.

In the present method, the effective zero-point is determined by a linear fitting of the contact stiffness values after the stable contact point using Eq. (6), where the range is of 100–200 nm in this case. The intersection of the fitting line on the *x*-axis (*y* = 0) was determined as the effective zero-point for the sample. Figure 2b shows the depth dependence of ${E}_{IT}$ and ${H}_{IT}$ with the zero-point adjustment and with zero-point shifts of ± 10 and ± 20 nm. The stable modulus for the ${E}_{IT}$ and ${H}_{IT}$ evaluated with the effective zero-point are the most stable among all the cases. The standard deviation of the ${E}_{IT}$, estimated as an average of the values at depth ranging from 80 to 2000 nm, becomes larger when the zero-point shift is 10 or 20 nm, as shown in Fig. 2c. It should be noted that the normal method produces a lower elastic modulus value with larger standard deviation in comparison with the present method.

### Examination of effective zero-point on pure metals

**Figure 3:**
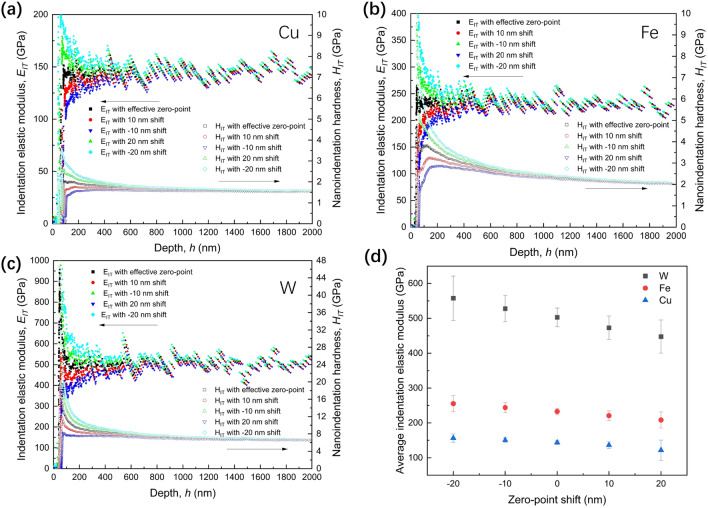
Impact of the zero-point shift on indentation elastic modulus and nanoindentation hardness on pure metals Cu, Fe, and W. (**a**) Indentation elastic modulus and nanoindentation hardness after zero-point shift on Cu. (**b**) Indentation elastic modulus and nanoindentation hardness after zero-point shift on Fe. (**c**) Indentation elastic modulus and nanoindentation hardness after zero-point shift on W. (**d**) Average and standard deviation of indentation elastic modulus after zero-point shift on Cu, Fe, and W.

Based on the area function obtained by the present method, the indentation elastic modulus and nanoindentation hardness were also evaluated for pure metallic materials, such as Cu, Fe and W, as shown in Fig. 3, which exhibited stable indentation elastic modulus in the wide range of indentation depth after the zero-point adjustment of the present method. Additionally, the indentation elastic modulus stabilizes beyond a depth of approximately 60 nm, demonstrating the validity of the nanoindentation test at 60 nm onwards. The zero-point was shifted by ± 10 and ± 20 nm from the effective zero-point to examine the zero-point assumption further. The non-shifted zero-point case result (black data points in Fig. 3a–c) demonstrated the best stability of the data among all cases, even within the shallow region. The ${E}_{IT}$ with the effective zero-point exhibits the most stable value, as shown in Fig. 3d.

### Impact of the zero-point shift on nanoindentation hardness

**Figure 4:**
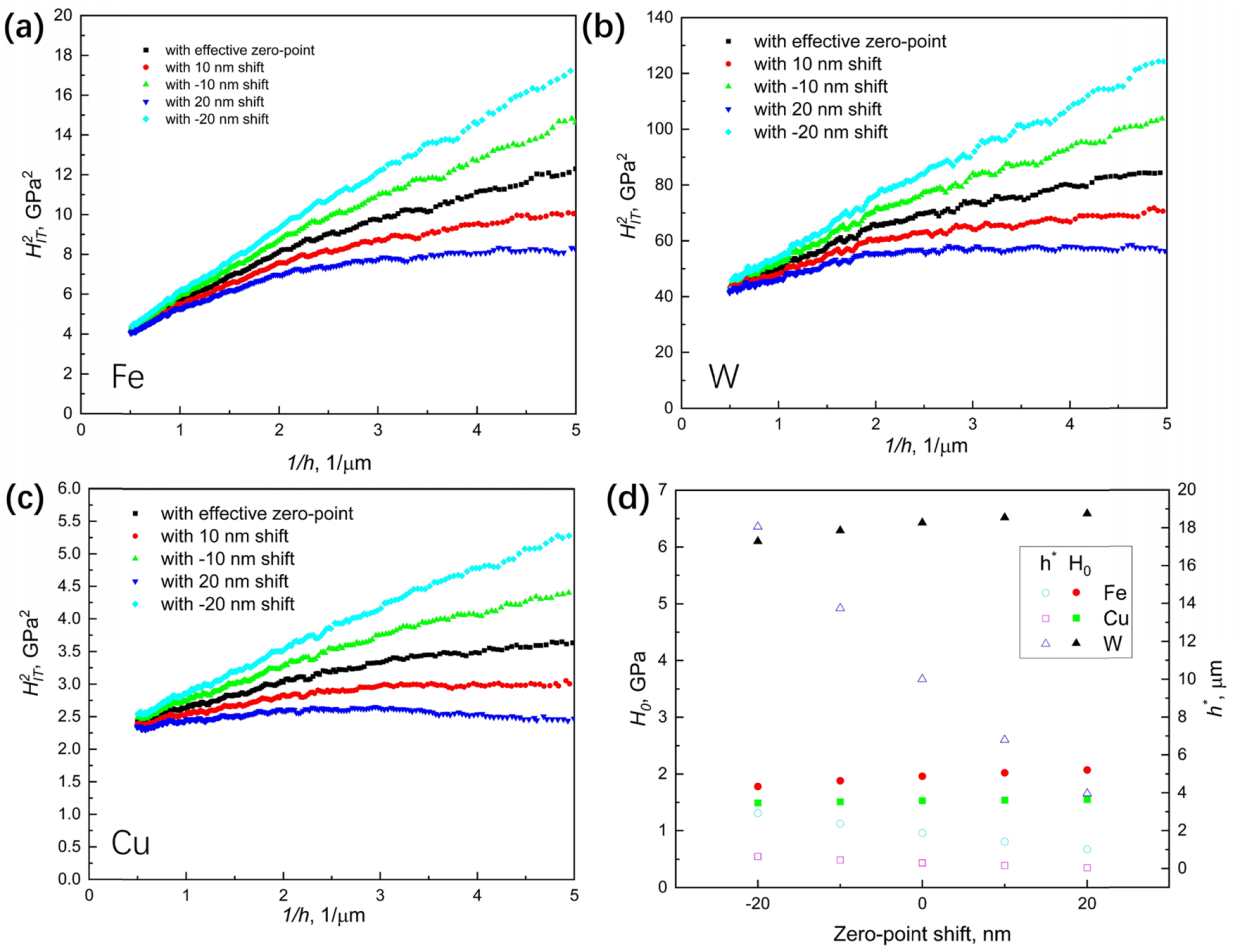
Influence of the zero-point shift on nanoindentation hardness. (**a**) Indentation size effect after zero-point shift on pure Fe. (**b**) Indentation size effect after zero-point shift on pure W. (**c**) Indentation size effect after zero-point shift on pure Cu. (**d**) ${H}_{0}$ and ${h}^{*}$ in Nix–Gao model after zero-point shift.

The zero-point shift severely affected the nanoindentation hardness estimates, as shown in Fig. 3a–c. By incorporating the results of the zero-point shift in Eq. (8), it can be shown that the influence of zero-point on hardness increases in significance as the depth decreases. Consequently, the ISE described in the Nix–Gao plot is significantly affected by the zero-point shift, as shown in Fig. 4a–c. Note that the characteristic length ${h}^{*}$ for the ISE and the linearity of the Nix–Gao plot are both significantly affected by the zero-point shift, whereas the bulk-equivalent nanoindentation hardness ${H}_{0}$ is not sensitive to the zero-point shift, as shown in Fig. 4d.

## Discussion

This study proposes a practical zero-point adjustment to optimise the nanoindentation tests using the CSM method with a Berkovich tip; furthermore, it addresses the impact of the zero-point shift on nanoindentation results, especially on the nanoindentation hardness. The present method simplifies the estimation of the point of contact between the tip and the sample surface, and provides a quick and reliable way to analyse nanoindentation test results. The indentation elastic modulus demonstrated a stable value against the indentation depth after the zero-point adjustment, and the nanoindentation hardness was also corrected using a reasonable effective zero-point assumption. It was observed that varying the zero-point shift by a few nanometres significantly affects the indentation elastic modulus and nanoindentation hardness at shallow depths.

In the normal method, the zero-point is determined based on the stiffness criterion of 200 N/m or any other input value; the tip has already penetrated the sample at this point, as shown in Fig. 1. As Fig. 2a suggests, the linear relationship between elastic modulus and stiffness fails when the zero-point is estimated by the normal method, which contradicts the assumption of constant elastic modulus of indented material against varying depth. In addition, it is obvious that the zero-point cannot be uniquely determined by this criterion. The shift of possible zero-points determined by stiffness criterion can be easily higher than the 10 or 20 nm in the present analysis, which introduces a much larger error. In contrast, the effective zero-point proposed in the present method has no conflict with the above assumption because it ignores the initial range of depth where the contact is not reliable in the CSM method. Hence, the proposed method can offer a reasonable effective zero-point based on the linear relationship between the stiffness and indentation depth.

The determination based on the Hertzian contact region (the first few dozen nanometres) was not always reliable because of the sinusoidally harmonic nature of the applied load in the CSM method. The effective zero-point estimation in the present method has incomparable advantages such as the determination of the zero-point from the stable bulk test rather than from the unstable surface contact. The experiments with standard fused silica and pure metals showed that the results with the effective zero-point were well optimised with better quality on the stability of the indentation elastic modulus and reasonable nanoindentation hardness value, especially within the shallow depths.

Researchers commonly use nanoindentation tests at less than 300 $\mathrm{nm}$ to investigate the mechanical properties of surface region^24,25^. Based on Eq. (8), the 10-nm zero-point shift could lead to more than 6% deviation in the nanoindentation hardness outcome. Moreover, severe depths, such as 100–150 nm, have been used to provide a surface analysis^26,27^, where the 10-nm zero-point shift might have contributed to a hardness deviation of higher than 23%. Both are fatal errors in the surface analysis. However, it has not received enough attention—the zero-point determination and relevant information have not been treated thoroughly. This problem can be effectively controlled using the CSM method with the proposed zero-point adjustment.

In addition, zero-point shift influences the ISE analysis. The ${H}_{0}$ remains effectively invariant after the shift owing to the contribution of hardness from the deep region. Currently, we observe that the Nix–Gao model of Eq. (9) is not perfect, and the linearity of the Nix–Gao plot highly depends on the materials used. As shown in the present study, the artefact caused by the zero-point shift can hugely affect the depth-dependence of the Nix–Gao model. The accuracy and linearity of the model are extremely sensitive to the zero-point shift. The effect of the zero-point shift must be considered to interpret the indentation size effect.

We believe that the present determination and adjustment using an effective zero-point is essential in nanoindentation tests because it offers an uncomplicated but efficient way to process the tests, which will become increasingly attractive with the increasing requirement for nanoindentation.

## Methods

Fused silica with an elastic modulus of 74 GPa—a standard sample in nanoindentation tests—was used to calibrate the tip area function. Cu (purity > 99.9 wt%), Fe (purity > 99.5 wt%), and W (purity > 99.7 wt%) were used to compare the effect of zero-point adjustment on different elastic–plastic materials. Before the nanoindentation test, the surfaces of the samples were polished to 40 nm with colloidal silica using a vibratory polisher to avoid surface defects. Nanoindentation tests were performed using a Nano Indenter G200 (Agilent Technologies) via the CSM method with a modified Berkovich tip. At least ten nanoindentations with a depth limited to 2000 nm, a frequency of 45 Hz, a harmonic amplitude of 1 nm, and a strain rate of 0.05 s^-1^ were performed on each sample. The actual area function of the Berkovich indenter was calibrated on the fused silica.

## Data Availability

The datasets generated or analysed during the current study are available from the corresponding author upon reasonable request.
